# Educational Attainment and Criminal Justice: A Nationwide Cohort Study of 4.3 Million Young People

**DOI:** 10.1007/s40865-026-00309-9

**Published:** 2026-06-30

**Authors:** Alice Wickersham, Rosie Cornish, Hannah Dickson, Stephen Scott, Johnny Downs

**Affiliations:** 1 CAMHS Digital Lab, Department of Child and Adolescent Psychiatry, King’s Maudsley Partnership, Institute of Psychiatry, Psychology & Neuroscience, King’s College London, London, United Kingdom; 2 Division of Psychiatry, University College London, London, United Kingdom; 3 South London and Maudsley NHS Foundation Trust, London, United Kingdom; 4 Population Health Sciences, Bristol Medical School, University of Bristol, Bristol, United Kingdom; 5 MRC Integrative Epidemiology Unit, University of Bristol, Bristol, United Kingdom; 6 Department of Forensic & Neurodevelopmental Sciences, Institute of Psychiatry, Psychology & Neuroscience, King’s College London, London, United Kingdom; 7 Department of Child and Adolescent Psychiatry, King’s Maudsley Partnership, Institute of Psychiatry, Psychology & Neuroscience, King’s College London, London, United Kingdom

**Keywords:** Education, School, Criminal justice, Longitudinal study, Trajectory modelling

## Abstract

Understanding predictors of offending is essential for developing effective crime prevention. Educational attainment is a readily available metric for pupils in many countries, with performance on standardised tests often systematically collected throughout school. If changing attainment is associated with offending risk, it could present a signal to check in with pupils and offer appropriate support. We analysed an existing linkage between the National Pupil Database and the Police National Computer in England. In a cohort of 4.3 million pupils born between the academic years 1990/91 and 1996/97, we modelled trajectories of standardised attainment on statutory tests at ages 7, 11 and 16 years. We then investigated the association between these attainment trajectories and subsequent first offence convictions or cautions during young adulthood. Among pupils showing relative declines in attainment over their school career, 1 in 3 were convicted or cautioned for a first offence before the end of school, and 1 in 10 were convicted or cautioned for a first offence during young adulthood. Additionally, among pupils who performed at below average levels towards the beginning of school, their odds of offending during young adulthood were 53% higher than average attainers if their relative attainment remained low, but only 4% higher if their relative attainment improved. Noticing relative changes in attainment could provide an important opportunity to offer appropriate support and prevent engagement in offending behaviour, mitigating subsequent criminal justice involvement and related public health impacts.

**Supplementary Information:**

The online version contains supplementary material available at 10.1007/s40865-026-00309-9.

## Introduction

Criminal offending and re-offending comes at a significant social and economic cost to society, and presents a serious public health concern (Heeks et al., 2018). Violence is estimated to cost the NHS £2.9 billion every year (Department of Health & Social Care, 2012), and incarcerated offenders experience high rates of death due to suicide and overdose post-release (Borschmann, Mortality After Release from Incarceration Consortium (MARIC) collaborators, & Kinner, 2024). Understanding risk and protective factors for criminal offending is therefore a key research priority for crime prevention policies, which would minimise the number of people entering the criminal justice system, and be a critical component of tackling related public health issues (Ministry of Justice, 2020).

‘School failure’ is one of many factors thought to increase long-term potential for antisocial behaviour (Farrington, 2019). Research shows associations between lower attainment and subsequent delinquency, offending, and criminal justice involvement (both life-course-persistent and adolescent-limited offending) (Basto-Pereira & Farrington, 2022; Jolliffe et al., 2017; Lankester et al., 2025; Savage et al., 2017). This association could be linked to various mechanisms thought to underlie offending onset. For example, consistent with Merton’s strain theory, educational failure could intensify the mismatch between desired cultural goals (such as wealth and status) and the means available to achieve them, leading individuals to experience strain and to consider other methods of achieving goals, such as offending (Farnworth & Leiber, 1989). Similarly, Sampson & Laub’s age-graded theory of informal social control suggests that weak social bonds with school and associated low attainment may limit the availability of “turning points” (such as employment) which would otherwise lead someone to desist from antisocial behaviour (Sampson & Laub, 1993).

Research suggests that educational factors like attainment may not be the strongest predictors of offending outcomes, with one overview of meta-analyses finding that family and parental factors play a stronger role (Basto-Pereira & Farrington, 2022). But crucially, attainment could be one of the most practically useful metrics for identifying intervention opportunities. Firstly, it is a readily available metric: performance on standardised tests is systematically collected at a national level for all pupils in England’s state schools (Jay et al., 2019), with many conducting additional non-statutory tests on a regular basis. Secondly, attainment is a sensitive indicator of not just individual cognitive ability (Cornish et al., 2015), but also environmental risks like family difficulties, mental health, or other school difficulties (Noltemeyer et al., 2015; Pinquart, 2016; Wickersham et al., 2021). Taking these points together, changing attainment presents a readily available signal to check in with pupils and offer support which, if tailored appropriately to their needs, could in turn mitigate risk for later outcomes like offending.

The dynamic nature of offending has long been recognised in criminology, with the long-established age-crime curve showing that criminal behaviour generally peaks in adolescence, and Moffitt’s dual-taxonomy theory further distinguishing between life-course persistent and adolescent-limited antisocial behaviour (Moffitt, 1993). And yet, many studies in this area do not analyse attainment as a dynamic construct (Lankester et al., 2025). Understanding changes in attainment over time would highlight early risk indicators and key intervention timepoints. Beyond survey data from small international cohorts which were unable to account for a range of potential confounders, little is known about how changes in attainment throughout school might be associated with offending (Nocentini et al., 2012). Findings from such studies are also equivocal, with some finding consistently low or declining achievement to be associated with higher delinquency (DuPaul et al., 2018; Zhang & Slesnick, 2020), but another finding no such association (Fleming et al., 2010). It also remains unclear whether certain attainment patterns differentially exacerbate or mitigate criminal offending risk in different schools and communities (thought to play a powerful role in whether young people enter the criminal justice system) (Jolliffe et al., 2017), or among known vulnerable groups (such as pupils from deprived backgrounds or in the care system) (Department for Education & Ministry of Justice, 2022). Understanding whether and how these risk factors interact with attainment patterns would help identify particularly vulnerable groups for crime prevention strategies.

Therefore, we aimed to investigate the association between educational attainment trajectories and criminal justice involvement. We focused on first offence convictions and cautions during young adulthood to ensure that the attainment trajectories preceded the outcome of interest, but did also explore the possible role of earlier offending. We sought to answer the following research questions:

1. Is attainment trajectory associated with subsequent criminal justice involvement during young adulthood?
2. Does attainment trajectory modify risk of criminal justice involvement during young adulthood among vulnerable and marginalised groups?
3. Does the association vary between schools and local authorities?

We hypothesised that pupils with consistently low or declining attainment trajectories are more likely to be convicted or cautioned for criminal offences compared to pupils with consistently high or improving attainment trajectories. We also hypothesised that offending risk among pupils who generally show higher rates of criminal justice involvement (males, certain ethnic groups, deprived pupils, pupils in care, and pupils with special educational needs) would be attenuated if they have consistently high or improving attainment trajectories.

## Methods

Analyses were pre-registered on the Open Science Framework (Wickersham, 2022a, b). Reporting follows guidelines for cohort studies using linked data, and for latent trajectory studies (Table S1.1, Table S1.2).

### Design and Sample

This was an historical, longitudinal cohort study using an existing individual-level data linkage between the National Pupil Database (NPD) and the Police National Computer (PNC). Linkage was undertaken by the ‘Data First’ programme, fully described elsewhere (Dillon et al., 2022). Approved researchers have access through a secure research server to de-identified NPD data for all pupils in England’s state schools, as well as linked crime data for a subset of these pupils who have a PNC record.

We used NPD records and (where applicable) PNC records for pupils born during the academic years 1990/91 and 1996/97. These birth cohorts were selected to maximise availability of our attainment and primary outcome variables. The NPD contains attainment data at three key timepoints when statutory testing takes place in England: Year 2 (key stage 1, typically assessed at age 6 or 7), Year 6 (key stage 2, age 10 or 11) and Year 11 (key stage 4, age 15 or 16). We excluded pupils who did not have attainment data for at least two of the three assessment timepoints to minimise risk of trajectory misclassification. A resulting n = 4,317,436 were included in analysis (Figure S2.1).

### Measures

#### Educational Attainment

We derived mean average point scores for each pupil, indicative of attainment on National Curriculum assessments in Year 2 (averaged across reading, writing, maths and science assessments, rounded to one decimal place) and Year 6 (averaged across English and maths assessments, rounded to one decimal place). At Year 11, we used a total point score variable which is based on each pupil’s General Certificate of Secondary Education or equivalent assessments. If pupils had multiple attainment records available for one timepoint, we kept the earliest record in which they performed their highest. Scores at each timepoint were then standardised to z-scores using the mean and standard deviation of all pupils in the sample who had attainment data at that timepoint, conducted separately for each academic year group to account for changes in grading over time.

#### Primary Offending Outcome: Any First Offence Caution or Conviction During Young Adulthood

Using the PNC, we derived a binary indicator of whether pupils went on to be convicted or cautioned for any first offence after completing secondary school (defined as 31 st August of their Year 11 assessment year), and up to and including age 21. All valid Home Office offence groups were included: violence against the person, sexual offences, robbery and theft offences, criminal damage and arson, drug offences, possession of weapons, public order offences, miscellaneous crimes against society, fraud offences, summary non-motoring offences, and summary motoring offences. Because we were primarily investigating first offence convictions or cautions during young adulthood, the reference group for this variable comprised all other pupils in the sample (including those with earlier first offences). However, for descriptive exploration and sensitivity analyses, we also derived a binary indicator of whether pupils were convicted or cautioned for any earlier first offences (i.e. before completing secondary school).

#### Secondary Offending Outcome: First Offence Caution or Conviction During Young Adulthood for Serious Violence

Using the PNC, we derived a binary indicator of whether pupils went on to be convicted or cautioned for any first offence after the end of Year 11 and up to and including age 21, where that first offence was a serious violence offence. This was done according to an existing definition of serious violence (Department for Education & Ministry of Justice, 2022), which mostly encompasses the following Home Office offence groups: violence against the person offences, robbery offences, and possession of weapons offences. As with the primary outcome, the reference group for this variable therefore comprised all other pupils in the sample, including those whose first offences were not for serious violence.

#### Covariates and Contextual Variables

Covariates were derived from the NPD, and included gender, ethnicity, free school meal (FSM) eligibility, special educational needs (SEN) status, Year 11 looked after child (LAC) status, and Year 11 assessment year. Year 11 assessment year was defined as the calendar year in which key stage 4 was completed (where missing, this was calculated by adding five to the Year 6 assessment year). For LAC status, we identified pupils as LAC if their records successfully linked to the NPD’s Children Looked After return in their Year 11 assessment year. All remaining covariates were ascertained from pupils’ most recently available school year to Year 11. For SEN status, we derived categories used in a previous publication: receiving lower levels of provision in the form of action, action plus or support (AAP/S), having a statement of SEN or an education, health and care plan (S/EHCPs), or neither (Jay & Gilbert, 2021).

School and local authority were used as contextual variables. They were derived from an NPD variable which combines a numeric code representing the establishment number of the school attended, and a numeric code representing the local authority that the school reports to. They were ascertained from pupils’ most recently available school year to Year 11.

### Statistical Analyses

#### Trajectory Modelling

Trajectory modelling of attainment z-scores over Years 2, 6 and 11 was conducted under a Structural Equation Modelling framework in Mplus version 8.7 (Berlin et al., 2013). Full details of the trajectory modelling process can be found in Data Supplement S2.1 and Table S2.1. Briefly, we derived a single-trajectory linear growth model which served as the base model for Linear Growth Mixture Modelling (LGMM). During LGMM, we fitted one to five latent trajectories, and selected the optimal number of trajectories to be accepted as the final model solution taking into account Akaike Information Criterion (AIC), Bayesian Information Criterion (BIC), Sample size-adjusted Bayesian Information Criterion (ABIC), classification tables, entropy, trajectory shapes and sizes, and advisory group insights from a previous study (Wickersham et al., 2023).

#### Multilevel Logistic Regression Modelling

Attainment trajectory membership was used as the main exposure variable in multilevel logistic regressions with primary and secondary outcomes (Research Question 1). First, an empty single-level model containing only the outcome variable was fitted. Two-level (pupil, school) and three-level (pupil, school, local authority) variance components models were then fitted and compared. After choosing the optimal number of levels to include in analysis (two-level, including pupil and school), we fitted random intercept models, adding in attainment trajectory membership as the main exposure variable, followed by the remaining covariates.

For the primary outcome, we tested interactions between attainment trajectory membership and each sociodemographic characteristic (Research Question 2). We also investigated the extent to which the association between attainment trajectory membership and the primary outcome varied at the level of school and local authority (Research Question 3). Due to limited computational power of the research server, these random slope models were restricted to a random subsample of *n* = 20 local authorities, and local authority and school clusters were investigated in separate two-level models. Multilevel logistic regressions were conducted in Stata version 17.0.

#### Missing Data

Missing data were investigated prior to trajectory modelling, and again prior to multilevel logistic regression modelling. In our sample of *n* = 4,317,436, attainment z-scores were missing for *n* = 220,197 (5.1%) in Year 2, *n* = 101,393 (2.4%) in Year 6, and *n* = 106,841 (2.5%) in Year 11. In total, *n* = 3,889,005 (90.1%) had attainment z-scores available for all three timepoints, and *n* = 428,431 (9.9%) for two timepoints. We found that missing a z-score at one timepoint was associated with the z-scores at the remaining two non-missing timepoints (Table S2.2), supporting a missing at random assumption for these data, and informing our use of Full Information Maximum Likelihood for trajectory modelling. Of the *n* = 4,317,436 included in trajectory modelling, covariate data was complete for *n* = 4,218,611 (97.7%) pupils (Table S2.3). Complete cases were very similar to the full sample on the remaining, non-missing variables, informing our use of complete case analysis for multilevel logistic regressions (Table S2.4).

## Results

**Fig. 1:**
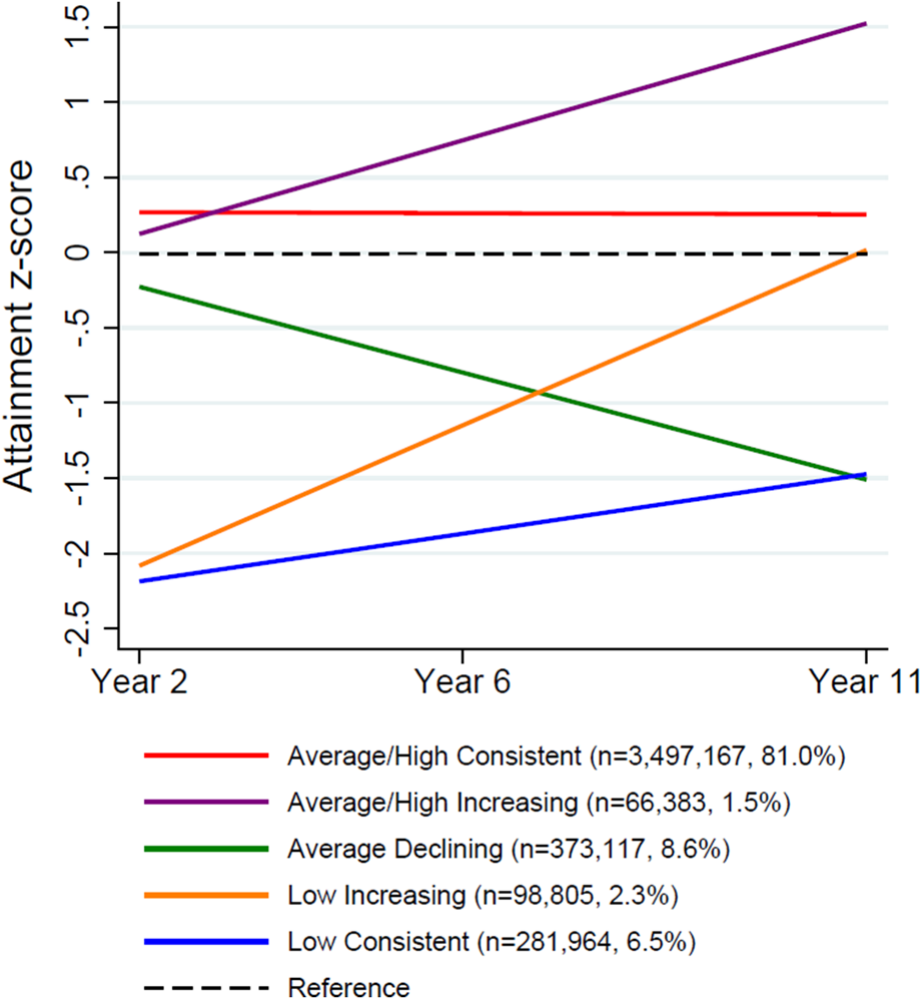
Five-trajectory LGMM solution

We modelled one to five attainment trajectories. Adding each trajectory improved overall model fit, with only moderate reductions in entropy and classification accuracy (Table 1). Each trajectory also represented a distinct pattern of relative change, culminating in different levels of attainment in Year 11 (Figure S3.1, Table S3.2, Table S3.3). Consistent with advisory group feedback that we received on a similar analysis (Wickersham et al., 2023), we therefore accepted the 5-trajectory LGMM solution (Fig. 1).

**Table 1:** Model fit for LGMM of educational attainment

Goodness-of-fit	1 trajectory	2 trajectories	3 trajectories	4 trajectories	5 trajectories
Number of free parameters	8	11	14	17	20
AIC	30333579.4	29769406.1	29618431.7	29558525.1	29512614.2
BIC	30333685.6	29769552.2	29618617.6	29558750.8	29512879.8
ABIC	30333660.2	29769517.2	29618573.1	29558696.8	29512816.2
Entropy	N/A	0.885	0.803	0.826	0.816
Classification accuracy range	N/A	0.868 to 0.978	0.757 to 0.942	0.691 to 0.940	0.700 to 0.920
Trajectory size (%)					
Trajectory 1	4,317,436 (100%)	3,948,905 (91.4%)	3,623,666 (83.9%)	3,593,112 (83.2%)	3,497,167 (81.0%)
Trajectory 2	-	368,531 (8.5%)	359,786 (8.3%)	71,158 (1.6%)	281,964 (6.5%)
Trajectory 3	-	-	333,984 (7.7%)	343,136 (7.9%)	373,117 (8.6%)
Trajectory 4	-	-	-	310,030 (7.2%)	98,805 (2.3%)
Trajectory 5	-	-	-	-	66,383 (1.5%)

Abbreviations: *ABIC* sample size-adjusted Bayesian information criterion, *AIC* Akaike information criterion, *BIC* Bayesian information criterion, *N/A* not applicable

Characteristics of the *n* = 4,218,611 included in multilevel logistic regression are in Table 2 (stratified by attainment trajectory in Table S4.1). First offence convictions and cautions during school and young adulthood were most frequently observed among males, FSM eligible pupils, LAC pupils in Year 11, and pupils in receipt of SEN provision (Table 3). Notably, 1 in 3 pupils showing declining relative attainment over their school career were convicted or cautioned for a first offence before the end of school, and 1 in 10 were convicted or cautioned for a first offence during young adulthood – higher rates than in other attainment trajectories.

**Table 2:** Sample characteristics, *n* = 4,218,611

	*n* (%)
Any first offence conviction or caution before the end of Year 11	369,557 (8.8%)
Any first offence conviction or caution during young adulthood	210,936 (5.0%)
First offence conviction or caution during young adulthood for serious violence	19,017 (0.5%)
Attainment trajectory membership	
*Average/High Consistent*	3,402,510 (80.7%)
*Average/High Increasing*	66,045 (1.6%)
*Average Declining*	370,319 (8.8%)
*Low Increasing*	98,568 (2.3%)
*Low Consistent*	281,169 (6.7%)
Gender	
*Female*	2,060,235 (48.8%)
*Male*	2,158,376 (51.2%)
Ethnic group	
*White*	3,574,486 (84.7%)
*Black*	161,886 (3.8%)
*Asian*	307,963 (7.3%)
*Mixed*	135,811 (3.2%)
*Other*	38,465 (0.9%)
FSM eligibility	
*Ineligible*	3,654,127 (86.6%)
*Eligible*	564,484 (13.4%)
LAC in Year 11	
*No*	4,168,510 (99.0%)
*Yes*	50,100 (1.0%)
SEN provision	
*AAP/S*	810,680 (19.2%)
*S/EHCPS*	167,662 (4.0%)
Year 11 assessment year (median, IQR)	2009 (2007 to 2011)

In accordance with Children Looked After statistical disclosure control guidelines, LAC status frequencies have been rounded to the nearest 10, and percentages to the nearest integer

Abbreviations: *AAP/S* action, action plus or support, *FSM* free school meals, *LAC* looked after child, *IQR* interquartile range, *S/EHCPS* statement of SEN or an education, health and care plan, *SEN* special educational needs

**Table 3:** Outcomes stratified by sample characteristics

	Any first offence conviction or caution before the end of secondary school	Any first offence conviction or caution during young adulthood (primary outcome)	First offence conviction or caution during young adulthood for serious violence (secondary outcome)
Attainment trajectory membership			
*Average/High Consistent*	181,460 (5.3%)	144,059 (4.2%)	12,235 (0.4%)
*Average/High Increasing*	1,122 (1.7%)	1,012 (1.5%)	69 (0.1%)
*Average Declining*	127,604 (34.5%)	36,332 (9.8%)	3,570 (1.0%)
*Low Increasing*	6,529 (6.6%)	5,517 (5.6%)	604 (0.6%)
*Low Consistent*	52,842 (18.8%)	24,016 (8.5%)	2,539 (0.9%)
Gender			
*Female*	119,545 (5.8%)	47,825 (2.3%)	3,009 (0.1%)
*Male*	250,012 (11.6%)	163,111 (7.6%)	16,008 (0.7%)
Ethnic group			
*White*	318,489 (8.9%)	174,764 (4.9%)	13,808 (0.4%)
*Black*	17,611 (10.9%)	12,571 (7.8%)	2,071 (1.3%)
*Asian*	14,012 (4.6%)	12,861 (4.2%)	1,779 (0.6%)
*Mixed*	17,022 (12.5%)	8,740 (6.4%)	1,087 (0.8%)
*Other*	2,423 (6.3%)	2,000 (5.2%)	272 (0.7%)
FSM eligibility			
*Ineligible*	268,339 (7.3%)	168,523 (4.6%)	14,262 (0.4%)
*Eligible*	101,218 (17.9%)	42,413 (7.5%)	4,755 (0.8%)
LAC status in Year 11			
*Not LAC*	347,760 (8.0%)	207,040 (5.0%)	18,600 (k)
*LAC*	21,800 (44.0%)	3,900 (8.0%)	420 (1.0%)
SEN provision			
*None*	179,819 (5.6%)	133,597 (4.1%)	11,111 (0.3%)
*AAP/S*	158,044 (19.5%)	66,182 (8.2%)	6,801 (0.8%)
*S/EHCPS*	31,694 (18.9%)	11,157 (6.7%)	1,105 (0.7%)
Year 11 assessment year (median, IQR)	2008 (2007 to 2010)	2008 (2007 to 2010)	2008 (2007 to 2010)

In accordance with Children Looked After statistical disclosure control guidelines, LAC status frequencies have been rounded to the nearest 10, and percentages to the nearest integer. ‘k’ is used when a result that is not 100%/0% would appear as such due to rounding

Abbreviations: *AAP/S* action, action plus or support, *FSM* free school meals, *LAC* looked after child, *IQR* interquartile range, *S/EHCPS* statement of SEN or an education, health and care plan, *SEN* special educational needs

In multilevel regression modelling, school-level clustering accounted for 5.9% of the variance in our primary outcome, whereas local authority-level clustering accounted for only 0.6%, such that omitting local authority-level clustering made no impact on estimated effects of attainment trajectory membership (Table S4.2). Similar findings emerged for our secondary outcome (Table S4.3). Therefore, the multilevel models reported here only include school-level clustering.

Findings from regression modelling confirmed that, as compared to the Average/High Consistent attainment trajectory, pupils in the Average/High Increasing trajectory had reduced odds for first offence convictions and cautions during young adulthood, whereas pupils in the Average Declining, Low Consistent, and (to a lesser degree) the Low Increasing trajectories had increased odds for this outcome (Table 4). Adjusting for sociodemographic variables slightly attenuated all associations, but the direction and pattern of findings remained the same. Notably, among pupils who performed at below average levels towards the beginning of school, their odds of first offences during young adulthood were 53% higher than average attainers if their attainment remained low relative to their cohort, but only 4% higher if their attainment improved relative to their cohort. A similar and slightly more pronounced pattern of results also emerged when focusing on first offence convictions and cautions during young adulthood which were serious violence offences (Table 4).

**Table 4:** Unadjusted and fully adjusted associations between attainment trajectory membership and (i) any first offence conviction or caution during young adulthood (primary outcome, left), (ii) first offence conviction or caution during young adulthood for serious violence (secondary outcome, right)

	Any first offence conviction or caution during young adulthood		First offence conviction or caution during young adulthood for serious violence	
	Unadjusted OR (95% CI)	Fully adjusted OR (95% CI)	Unadjusted OR (95% CI)	Fully adjusted OR (95% CI)
Fixed effects parameter estimates				
Intercept	0.04 (0.04 to 0.04)	0.03 (0.03 to 0.03)	0.00 (0.00 to 0.00)	0.00 (0.00 to 0.00)
Attainment trajectory membership				
*Average/High Consistent*	Reference	Reference	Reference	Reference
*Average/High Increasing*	0.33 (0.31 to 0.35)	0.39 (0.36 to 0.41)	0.26 (0.21 to 0.33)	0.32 (0.25 to 0.40)
*Average Declining*	2.30 (2.27 to 2.33)	1.78 (1.76 to 1.81)	2.50 (2.41 to 2.60)	1.82 (1.75 to 1.90)
*Low Increasing*	1.18 (1.15 to 1.22)	1.04 (1.01 to 1.07)	1.36 (1.25 to 1.48)	1.09 (1.00 to 1.18)
*Low Consistent*	2.04 (2.01 to 2.07)	1.53 (1.51 to 1.56)	2.29 (2.19 to 2.39)	1.60 (1.52 to 1.68)
Random effect: school variance	0.17 (0.16 to 0.18)	0.06 (0.05 to 0.06)	0.32 (0.29 to 0.35)	0.11 (0.10 to 0.13)
School ICC	4.8%	1.7%	8.8%	3.2%
Deviance	1,634,204	1,561,658	237,228	226,511

*CI* confidence interval, *ICC* intra class correlation, *OR* odds ratio. Pupil *n* = 4,218,611, school *n* = 19,554. Fully adjusted models included gender, ethnicity, FSM eligibility, LAC status in Year 11, SEN provision, and Year 11 assessment year

We tested for interactions between attainment trajectory membership and each sociodemographic variable (Table S4.4). Odds of our primary offending outcome were elevated across most attainment trajectories for males, Black and Mixed ethnic groups, pupils eligible for FSM, LAC pupils in Year 11, and pupils receiving AAP/S. But, contrary to our hypothesis, these increased odds were typically attenuated in low and declining trajectories (Table S4.5). Stratified analyses showed that the elevated offending odds associated with being male, Black or Mixed ethnicity, FSM eligibility, and receiving AAP/S were weakest in the Average Declining trajectory, as reflected by lower odds ratios in this trajectory compared to others. The stratified analyses suggested a more complex story for LAC status in Year 11; the Low Increasing and Average/High Consistent trajectories showed elevated odds of offending associated with being in care, whereas the Average Declining trajectory showed lower odds of offending associated with being in care.

We also investigated the extent to which the association between attainment trajectory membership and our primary offending outcome varied at the level of school and local authority. In random slope models, trajectory variances were consistently close to zero (Table S4.6 and Table S4.7), although at the school level there was more variability in the strength of association between some trajectories and the primary offending outcome.

### Supplementary Analyses

We conducted a sensitivity analysis to investigate the potential impact of including earlier offenders in the reference group of our primary outcome (Table S4.8). Findings were similar to the main analysis. However, excluding pupils who were convicted or cautioned for an offence before the end of secondary school from the reference group reversed the effect of the Low Increasing trajectory (although it remained weak, OR = 0.97, 95% CI = 0.94 to 0.99), while the effect of the Average Declining trajectory strengthened (OR = 2.78, 95% CI = 2.74 to 2.82). Effect modification findings were also similar after removing earlier offenders from the reference group (Table S4.9). A notable exception was in the Average Declining trajectory. In this trajectory, being in care in Year 11 was associated with reduced odds of offending in the main analysis, but increased odds when earlier offenders were excluded from the reference group.

Finally, we descriptively explored different offence types and repeat offences. Across different offence types, the likelihood of first offence conviction or caution during young adulthood was consistently highest in either the Average Declining or Low Consistent trajectories (Table S4.10). Summary motoring offences were perhaps the least differentiated by attainment trajectory. Additionally, among those convicted or cautioned for any first offence during young adulthood, the Average Declining and Low Consistent trajectories had the highest proportion of individuals with multiple offence occurrences on record (Table S4.11).

## Discussion

For the first time in a national cohort of pupils, we found that relative declines in attainment throughout school were most strongly associated with increased risk for first offences during young adulthood, followed by consistently low relative attainment. This pattern persisted for multiple offence types, and was even more pronounced for serious violence offences, which present a significant public health concern (Department of Health & Social Care, 2012).

We extend previous studies supporting associations between low attainment and offending by examining changes in attainment over time, rather than as a static construct (Basto-Pereira & Farrington, 2022; Farrington, 2019; Jolliffe et al., 2017; Lankester et al., 2025; Savage et al., 2017). Achievement is sometimes thought to be highly stable throughout school (and highly heritable) (Rimfeld et al., 2018), and while the majority of our sample indeed belonged to trajectories showing fairly consistent performance over time, a non-negligible 12.4% belonged to trajectories showing either increasing or decreasing relative performance. Their association with offending risk highlights the importance of treating attainment as a time-varying factor in research. Exploring educational attainment as a dynamic construct is perhaps particularly informative for the field of criminology, where the age-crime curve and differential onset, continuation, and desistence of offending has long been explored (Dickson, 2023; Moffitt, 1993). Although we primarily focused on first convictions or cautions during young adulthood, it is perhaps unsurprising that declining relative attainment through adolescence was most strongly associated with our outcome, plausibly coinciding with the emergence of antisocial behaviours that commonly onset during this period, and which could precede subsequent offending. A fruitful area for future research would be further exploring co-development of educational attainment and offending trajectories, to understand their relative timing.

Because administrative data are not primarily collected for research purposes, available covariates were necessarily limited, meaning that unmeasured confounding still precludes causal inference. For example, IQ, temperament, and parenting could play important roles which we were unable to assess. Findings from a recent sibling-based study are consistent with the possibility that attainment may exert a causal influence on criminal involvement (van de Weijer et al., 2024). However, regardless of whether or not the association we observed between attainment trajectory and offending is causal, our findings illustrate the potential for changes in attainment to clearly signal when pupils need additional support. If pupils are showing relative declines in their attainment, this presents an important opportunity to check in with those pupils to understand whether they need support, and if so, what the most appropriate support might be. This could also provide services with a window to initiate preventative interventions, although this would need to be carefully weighed against the costs of such interventions and possible risks, such as labelling effects (Bernburg et al., 2006). Additionally, among pupils performing below average in Year 2, their adjusted odds for young adult offending were 53% higher than consistently high/average attainers if they stayed on a low attainment trajectory, but only 4% higher if their attainment improved relative to their cohort. This shows further promise for the potential impact of offering additional support to below average attainers.

It should be acknowledged that the support some pupils need might not be predominantly educational. Consultation with a local Young Person’s Mental Health Advisory Group (YPMHAG) suggested that family problems, gang involvement, neighbourhood issues, homelessness, and experience of the care system might all be drivers of offending risk. These and other factors like verbal ability, impulsivity/hyperactivity, emotional lability, parenting style and social environment therefore present areas where pupils might be struggling and needing support. Pupils experiencing multiple risk factors for criminal offending might benefit from established crime prevention programs like Home Office Violence Reduction Units (Home Office, 2023), but the YPMHAG also highlighted the potential benefit of youth groups which engage with and provide a space for young people, without explicitly being for ‘crime prevention’ purposes.

Nonetheless, if educational support is required, schools might give struggling pupils the option to pursue alternative educational or vocational pathways which suit their skills and circumstances. This could offset some of the theoretical mechanisms underlying the relationship between attainment and offending, such as the mismatch between goals and means highlighted by Merton’s strain theory. Universal school-based socio-emotional learning programs have been found to both increase academic achievement and decrease conduct problems (Durlak et al., 2022), and early educational interventions also show some promise for preventing crime and associated financial costs to government and society (Paull & Xu, 2017). Future research could explore this possibility further by investigating whether existing interventions, like timely access to SEN provision, could mitigate offending risk among pupils who show early signs of struggling in school.

Contrary to our hypothesis, offending risk was attenuated among sociodemographic groups who generally show higher rates of criminal justice involvement if they had declining or consistently low attainment trajectories. This runs counter to the prevailing belief that, among those vulnerable groups, high attainment functions as a protective factor. However, it is potentially consistent with findings from the Cambridge Study in Delinquent Development that socioeconomic risk factors predict offending more strongly among females than males (Farrington & Painter, 2004). It is therefore plausible that for already-vulnerable groups like those explored in our study (such as males, Black and Mixed ethnic groups, and pupils eligible for FSM), additional effects of further disadvantage are less pronounced. Potentially, non-cognitive risk factors (such as motivation and socio-emotional regulation) supersede cognitive risk factors like academic achievement among these vulnerable groups (Heckman, 2008). However, further exploration around these unexpected findings would be needed before firm conclusions can be drawn.

School and local authority accounted for little variation in our outcome, suggesting that these factors may only be modestly criminogenic for offending that starts during young adulthood. The magnitudes of our school-level ICCs are generally consistent with those reported by other European studies using survey data and self-reported offending, which similarly show stronger contextual effects for violent offending (Higgins et al., 2020; Oberwittler, 2004; Pauwels et al., 2015). While prior studies characterise these ICCs as indicating relatively small school-level effects, they also highlight that this does not render the school context irrelevant to offending outcomes, and that multiple school-level contextual factors could nonetheless play a role in a young person’s offending outcomes. We suggest using the data linkage in this study to further explore contextual effects for offending which results in criminal justice involvement, building on existing multilevel analyses in these data which are specific to serious violence (Department for Education & Ministry of Justice, 2022). We also highlight that our random slope models were limited to 20 random local authorities due to limited computational power; therefore appropriate caution is warranted regarding these findings.

As with other administrative data linkage studies, some selection biases in this linkage could limit generalisability (Dillon et al., 2022). The justice records used here only capture data for individuals who commit criminal offences and are subsequently convicted or cautioned, while the education records only capture pupils in England’s state-funded schools. In Years 2 and 6, the Department for Education recommends that pupils who are absent from assessments or disapplied from the national curriculum are not assigned point score equivalents, such that these pupils are missing attainment data. Nonetheless, the majority of such pupils in this study had attainment data available at the remaining timepoints, and so were still captured in our trajectory models. Therefore, while caution is warranted in generalising our findings to such pupils, the impact of this is hopefully minimal. Additionally, our main analyses focused on first offenders during young adulthood, who are not necessarily representative of the wider offending population (we did so to highlight a direction of effect and draw inferences about potential prevention strategies). Nonetheless, administrative records remain advantageous in capturing whole populations, thereby minimising sampling and attrition biases which are common in surveys (Wickersham & Downs, 2023).

Further linkage to other administrative or survey datasets would enrich available data to explore possible mechanisms. For example, integrating health data which capture referral to Child and Adolescent Mental Health Services or Youth Offending Services would help us understand the role that mental health problems play in these relationships (Wickersham et al., 2023), and integrating household data with sibling links would help explore other vulnerabilities and possible confounding (van de Weijer et al., 2024). Social services and family court data would shed further light on engagement with other services, and whether these also present missed opportunities for intervention.

Overall, this study emphasises the need for timely support among pupils who are showing early signs of struggling in school, as these pupils may be at increased risk of experiencing difficulties like criminal justice involvement in young adulthood.

## Supplementary Information

Below is the link to the electronic supplementary material.

Supplementary file1 (DOCX 213 KB)

## Data Availability

The data cannot be made publicly available, but can be accessed with permissions from the Ministry of Justice and the Department for Education. The Department for Education extracted the data, which was deposited and accessed by AW in the Secure Research Service, part of the Office for National Statistics. Statistical code used for data analysis will be uploaded to the corresponding author’s GitHub upon publication (AW, https://github.com/AliceWickersham). For the purposes of open access, the author has applied a Creative Commons Attribution (CC BY) licence to any Accepted Author Manuscript version arising from this submission.
