# Supplementary material for: Educational Attainment and Criminal Justice: A Nationwide Cohort Study of 4.3 Million Young People
Source: J Dev Life Course Criminol. 2026 Jun 30;12(1):23. doi: 10.1007/s40865-026-00309-9 (PMC13348352; doi:10.1007/s40865-026-00309-9)
Supplement: Supplementary file 1 — Supplementary file1 (DOCX 213 KB) [file 40865_2026_309_MOESM1_ESM.docx]

Table of Contents

[Supplement 1 – Reporting checklists 3](#_Toc230344389)

[Table S1.1: STROBE Statement – Checklist of items that should be included in reports of cohort studies. 3](#_Toc230344390)

[Table S1.2: Guidelines for Reporting on Latent Trajectory Studies. 5](#_Toc230344391)

[Supplement 2 – Supporting information for methods 6](#_Toc230344392)

[Figure S2.1: Cohort flow diagram. 6](#_Toc230344393)

[Data Supplement S2.1: Trajectory modelling process. 7](#_Toc230344394)

[Table S2.1: Mean time between assessments in years. 9](#_Toc230344395)

[Table S2.2: Mean z-scores at each timepoint, stratified by availability of school performance data at the remaining timepoints. 9](#_Toc230344396)

[Table S2.3: Covariate availability across the total sample (n=4,317,436). 9](#_Toc230344397)

[Table S2.4: Characteristics of complete versus incomplete cases on fully available variables 10](#_Toc230344398)

[Supplement 3 – Supporting information for trajectory modelling findings 11](#_Toc230344399)

[Figure S3.1: Trajectory shapes for LGMM solutions with one to five latent trajectories. 11](#_Toc230344400)

[Table S3.2: Unstandardised parameter estimates for the five-trajectory solution. 12](#_Toc230344401)

[Table S3.3: Proportion of pupils meeting expected attainment thresholds for each school year in each trajectory, n (%). 12](#_Toc230344402)

[Supplement 4 – Supporting information for results 13](#_Toc230344403)

[Table S4.1: Sample characteristics stratified by attainment trajectory membership. 13](#_Toc230344404)

[Table S4.2: Single-level, variance-components, and multilevel models, with the outcome as any first offence conviction or caution during young adulthood (primary outcome). 14](#_Toc230344405)

[Table S4.3: Single-level, variance-components, and multilevel models, with the outcome as first offence conviction or caution during young adulthood for serious violence (secondary outcome). 15](#_Toc230344406)

[Table S4.4: Interaction terms between attainment trajectory membership and each sociodemographic covariate, with the outcome variable as any first offence conviction or caution during young adulthood. 16](#_Toc230344407)

[Table S4.5: Multilevel logistic regression models stratified by attainment trajectory membership, fully adjusted for all remaining covariates, and with the outcome as any first offence conviction or caution during young adulthood (primary outcome). 17](#_Toc230344408)

[Table S4.6: Two-level multilevel logistic regression models with random slopes for attainment trajectory membership at the level of local authorities, with the outcome as any first offence conviction or caution during young adulthood (primary outcome). 18](#_Toc230344409)

[Table S4.7: Two-level multilevel logistic regression models with random slopes for attainment trajectory membership at the level of schools, with the outcome as any first offence conviction or caution during young adulthood (primary outcome). 19](#_Toc230344410)

[Table S4.8: Sensitivity multilevel logistic regression analysis showing adjusted associations between attainment trajectory membership and any first offence conviction or caution during young adulthood (primary outcome), excluding pupils who were convicted or cautioned for an earlier offence before the end of secondary school from the reference group. 20](#_Toc230344411)

[Table S4.9: Sensitivity multilevel logistic regression models stratified by attainment trajectory membership, fully adjusted for all remaining covariates, and with the outcome as any first offence conviction or caution during young adulthood (primary outcome), excluding pupils who were convicted or cautioned for an earlier offence before the end of secondary school from the reference group. 21](#_Toc230344412)

[Table S4.10: Proportion of pupils in each attainment trajectory who were convicted or cautioned for different types of first offence during young adulthood. 22](#_Toc230344413)

[Table S4.11: Number of offending days in the period among those convicted or cautioned for different types of first offence during young adulthood, stratified by attainment trajectory. 22](#_Toc230344414)

# Supplement 1 – Reporting checklists

## Table S1.1: STROBE Statement – Checklist of items that should be included in reports of cohort studies.

|  | Item No | Recommendation | Page |
| --- | --- | --- | --- |
| **Title and abstract** | 1 | (*a*) Indicate the study’s design with a commonly used term in the title or the abstract | 1 |
|  |  | (*b*) Provide in the abstract an informative and balanced summary of what was done and what was found | 1 |
| Introduction | | | |
| Background/rationale | 2 | Explain the scientific background and rationale for the investigation being reported | 1-3 |
| Objectives | 3 | State specific objectives, including any prespecified hypotheses | 2 |
| Methods | | | |
| Study design | 4 | Present key elements of study design early in the paper | 3 |
| Setting | 5 | Describe the setting, locations, and relevant dates, including periods of recruitment, exposure, follow-up, and data collection | 3 |
| Participants | 6 | (*a*) Give the eligibility criteria, and the sources and methods of selection of participants. Describe methods of follow-up | 3 |
|  |  | (*b*) For matched studies, give matching criteria and number of exposed and unexposed | N/A |
| Variables | 7 | Clearly define all outcomes, exposures, predictors, potential confounders, and effect modifiers. Give diagnostic criteria, if applicable | 3-4 |
| Data sources/ measurement | 8* | For each variable of interest, give sources of data and details of methods of assessment (measurement). Describe comparability of assessment methods if there is more than one group | 3-4 |
| Bias | 9 | Describe any efforts to address potential sources of bias | 5 |
| Study size | 10 | Explain how the study size was arrived at | Supplement 2 |
| Quantitative variables | 11 | Explain how quantitative variables were handled in the analyses. If applicable, describe which groupings were chosen and why | 3-4 |
| Statistical methods | 12 | (*a*) Describe all statistical methods, including those used to control for confounding | 4-5 |
|  |  | (*b*) Describe any methods used to examine subgroups and interactions | 5 |
|  |  | (*c*) Explain how missing data were addressed | 5 |
|  |  | (*d*) If applicable, explain how loss to follow-up was addressed | N/A |
|  |  | (*e*) Describe any sensitivity analyses | 11 |
| Results | | | |
| Participants | 13* | (a) Report numbers of individuals at each stage of study—eg numbers potentially eligible, examined for eligibility, confirmed eligible, included in the study, completing follow-up, and analysed | Supplement 2 |
|  |  | (b) Give reasons for non-participation at each stage | Supplement 2 |
|  |  | (c) Consider use of a flow diagram | Supplement 2 |
| Descriptive data | 14* | (a) Give characteristics of study participants (eg demographic, clinical, social) and information on exposures and potential confounders | 8 |
|  |  | (b) Indicate number of participants with missing data for each variable of interest | Supplement 2 |
|  |  | (c) Summarise follow-up time (eg, average and total amount) | Supplement 2 |
| Outcome data | 15* | Report numbers of outcome events or summary measures over time | 9 |
| Main results | 16 | (*a*) Give unadjusted estimates and, if applicable, confounder-adjusted estimates and their precision (eg, 95% confidence interval). Make clear which confounders were adjusted for and why they were included | 10 |
|  |  | (*b*) Report category boundaries when continuous variables were categorized | N/A |
|  |  | (*c*) If relevant, consider translating estimates of relative risk into absolute risk for a meaningful time period | N/A |
| Other analyses | 17 | Report other analyses done—eg analyses of subgroups and interactions, and sensitivity analyses | 10-11 |
| Discussion | | | |
| Key results | 18 | Summarise key results with reference to study objectives | 11-14 |
| Limitations | 19 | Discuss limitations of the study, taking into account sources of potential bias or imprecision. Discuss both direction and magnitude of any potential bias | 13 |
| Interpretation | 20 | Give a cautious overall interpretation of results considering objectives, limitations, multiplicity of analyses, results from similar studies, and other relevant evidence | 11-14 |
| Generalisability | 21 | Discuss the generalisability (external validity) of the study results | 13 |
| Other information | | | |
| Funding | 22 | Give the source of funding and the role of the funders for the present study and, if applicable, for the original study on which the present article is based | 14 |

**Give information separately for exposed and unexposed groups.*

*Reference: von Elm, E., Altman, D. G., Egger, M., Pocock, S. J., Gøtzsche, P. C., & Vandenbroucke, J. P. (2007). The Strengthening the Reporting of Observational Studies in Epidemiology (STROBE) statement: guidelines for reporting observational studies. The Lancet, 370(9596), 1453-1457.*

## Table S1.2: Guidelines for Reporting on Latent Trajectory Studies.

| **#** | **Checklist Item** | **Page** |
| --- | --- | --- |
| 1 | Is the metric of time used in the statistical model reported? | 4 |
| 2 | Is information presented about the mean and variance of time within a wave? | Supplement 2 |
| 3a. | Is the missing data mechanism reported? | 5 |
| 3b. | Is a description provided of what variables are related to attrition/missing data? | 5 |
| 3c. | Is a description provided of how missing data in the analyses were dealt with? | 5 |
| 4 | Is information about the distribution of the observed variables included? | Supplement 2 |
| 5 | Is the software mentioned? | 4 |
| 6a. | Are alternative specifications of within-class heterogeneity considered (e.g., LGCA vs. LGMM) and clearly documented? If not, was sufficient justification provided as to eliminate certain specifications from consideration? | N/A^1^ |
| 6b. | Are alternative specifications of the between-class differences in variance/covariance matrix structure considered and clearly documented? If not, was sufficient justification provided as to eliminate certain specifications from consideration? | N/A^2^ |
| 7 | Are alternative shape/functional forms of the trajectories described? | Supplement 2 |
| 8 | If covariates have been used, can analyses still be replicated? | N/A^3^ |
| 9 | Is information reported about the number of random start values and final iterations included? | Supplement 2 |
| 10 | Are the model comparison (and selection) tools described from a statistical perspective? | 5-6 |
| 11 | Are the total number of fitted models reported, including a one-class solution? | 5-6 |
| 12 | Are the number of cases per class reported for each model (absolute sample size, or proportion)? | 6 |
| 13 | If classification of cases in a trajectory is the goal, is entropy reported? | 6 |
| 14a. | Is a plot included with the estimated mean trajectories of the final solution? | 6 |
| 14b. | Are plots included with the estimated mean trajectories for each model? | Supplement 3 |
| 14c. | Is a plot included of the combination of estimated means of the final model and the observed individual trajectories split out for each latent class? | N/A^4^ |
| 15 | Are characteristics of the final class solution numerically described (i.e., means, SD/SE, n, CI, etc.)? | Supplement 3 |
| 16 | Are the syntax files available (either in the appendix, supplementary materials, or from the authors)? | Data availability statement |

*^1^ LGMM was found to result in better model fit than LGCA in a previous study using these attainment data (Wickersham et al., 2023), so only LGMM was attempted in this study.*

*^2^ Alternative specifications of between-class differences were not specified a priori, so were not attempted.*

*^3^ No covariates were used in trajectory modelling.*

*^4^ Owing to statistical disclosure control regulations applied to these data, we are unable to display trajectories for individuals.*

*Reference: Van De Schoot R, Sijbrandij M, Winter SD, Depaoli S, Vermunt JK. The GRoLTS-checklist: guidelines for reporting on latent trajectory studies. Structural Equation Modeling: A Multidisciplinary Journal 2017; 24(3): 451-67.*

# Supplement 2 – Supporting information for methods

## Figure S2.1: Cohort flow diagram.

**
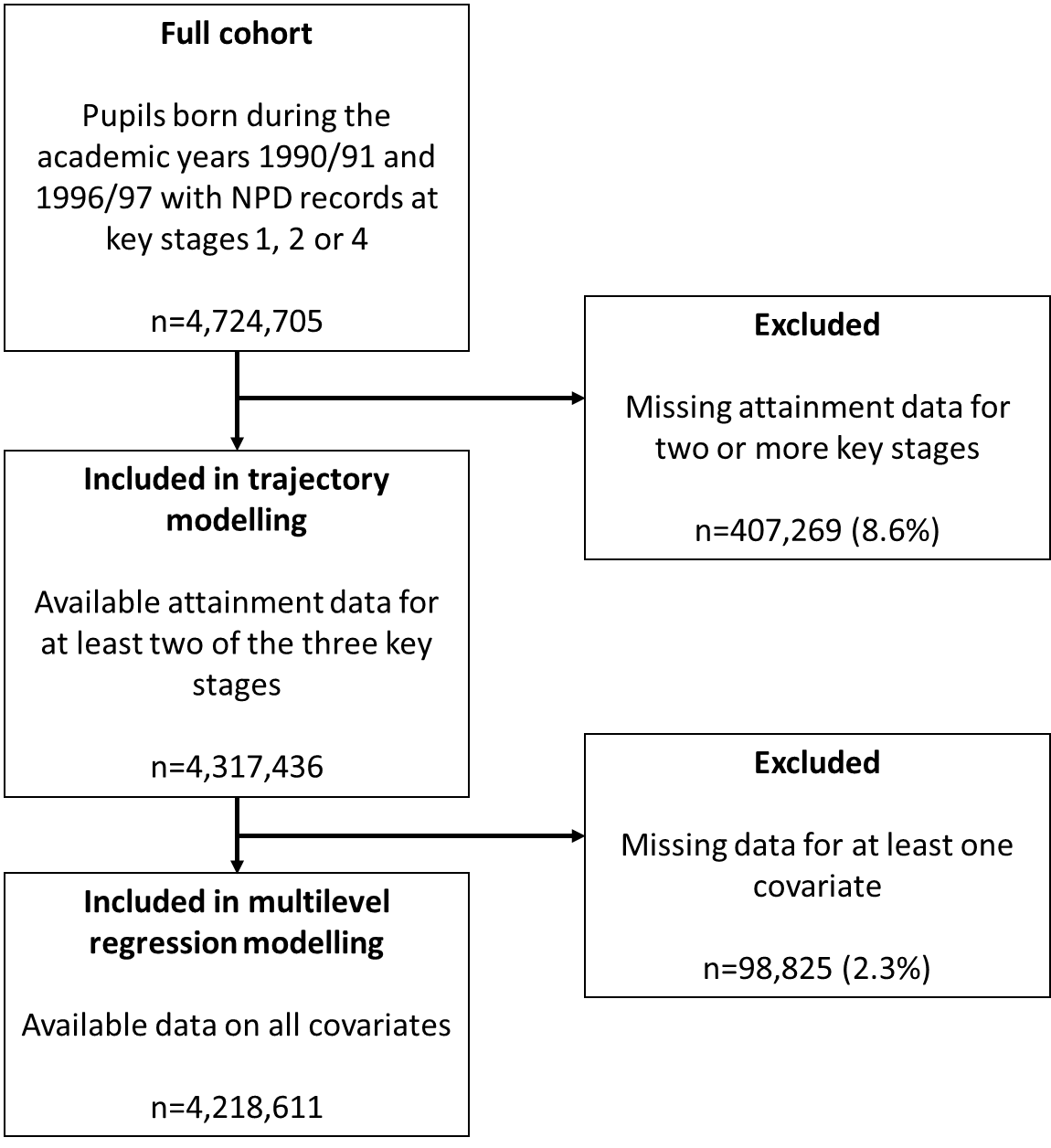
**

## Data Supplement S2.1: Trajectory modelling process.

Trajectory modelling of attainment z-scores over Years 2, 6 and 11 was conducted under a Structural Equation Modelling (SEM) framework in Mplus version 8.7, and followed a four-step procedure: problem definition, model specification, model estimation, and model selection and interpretation.

1. Problem definition

We developed a single-trajectory growth model to serve as the base model for subsequent modelling. Maximum likelihood estimation with robust standard errors was used, as attainment z-scores were continuous and skewed in each school year. Plotting trajectories for a random subsample of pupils indicated a tendency for linear trajectories, so we proceeded with linear growth modelling (LGM). Because some non-linear trajectories were observed, we also attempted piecewise growth models, but we encountered difficulties in the specification and convergence of these models, so reverted to LGM in accordance with our analysis plan. In LGM, the intercept factor loading was fixed to 1, and the slope factor loadings to 0, 4 and 9, reflecting the average number of years between each assessment timepoint (Table S2.1), and the intercept and slope were permitted to correlate.

Goodness-of-fit was evaluated using the following criteria for good model fit: Comparative Fit Index (CFI) ≥0.95, Tucker-Lewis Index (TLI) ≥0.95, Root Mean Square Error of Approximation (RMSEA) ≤0.06, and Standardized Root Mean Square Residual (SRMR) ≤0.08. While Chi-square (X^2^) is also reported, its p-value was not relied upon to assess goodness-of-fit, as this is heavily influenced by large sample sizes.

Linear growth modelling offered a good model fit (CFI=1.000, TLI=1.000, RMSEA=0.006, SRMR=0.001, X^2^(1)=152.4, p<0.001), and was accepted as the base model for Linear Growth Mixture Modelling (LGMM).

1. Model specification

Once the base models had been specified, we conducted LGMM, fitting one to five latent trajectories. Variances and covariances were constrained across latent trajectories (the default in Mplus for GMM), whereas intercept and slope means were freely estimated.^[[1]](#footnote-1)^

1. Model estimation

Models were fitted using the Mplus default of 20 random starts and 4 final stage optimisations. Random starts were then increased if necessary to replicate the best log-likelihood, and further increased to check for local solutions. We also checked for negative variances (Heywood cases).

1. Model selection and interpretation

We modelled one to five attainment trajectories, and attempted a sixth trajectory, but encountered residual covariance matrices which were not positive definite.

The optimal number of trajectories to be accepted as the final model solution was informed by the Akaike Information Criterion (AIC), Bayesian Information Criterion (BIC), and Sample size-adjusted Bayesian Information Criterion (ABIC) (for each of these fit statistics, models with lower values are favoured). We also took into account classification tables, entropy, trajectory shapes and sizes, and advisory group insights from a previous study.

For subsequent modelling, we derived most likely attainment trajectory membership for each pupil using individual-level class probabilities. Using most likely attainment trajectory membership as a categorical variable in subsequent regression models overlooks classification uncertainty; however, the high entropy observed in our final model suggested that classification accuracy was sufficiently high for this to present a minor concern.^[[2]](#footnote-2)^

## Table S2.1: Mean time between assessments in years.

|  | **Mean (SD)** |
| --- | --- |
| **Year 2 to Year 6** | 4.00 (0.05)  n=4,030,087 |
| **Year 6 to Year 11** | 5.01 (0.09)  n=4,149,246 |
| **Year 2 to Year 11** | 9.01 (0.10)  n=4,014,081 |

*Abbreviations: SD=Standard Deviation.*

## Table S2.2: Mean z-scores at each timepoint, stratified by availability of school performance data at the remaining timepoints.

|  | **Mean Year 2 z-score (SD)** | **Mean Year 6 z-score (SD)** | **Mean Year 11 z-score (SD)** |
| --- | --- | --- | --- |
| Year 2 z-score availability |  |  |  |
| *Available* | - | 0.01 (0.99)  n=3,995,846 | 0.00 (1.00)  n=3,990,398 |
| *Missing* | - | -0.19 (1.20)  n=220,197 | -0.01 (1.05)  n=220,197 |
| Year 6 z-score availability |  |  |  |
| *Available* | 0.00 (0.99)  n=3,995,846 | - | 0.01 (1.00)  n=4,109,202 |
| *Missing* | -0.10 (1.26)  n=101,393 | - | -0.34 (1.11)  n=101,393 |
| Year 11 z-score availability |  |  |  |
| *Available* | 0.01 (0.99)  n=3,990,398 | 0.01 (0.99)  n=4,109,202 | - |
| *Missing* | -0.50 (1.26)  n=106,841 | -0.49 (1.17)  n=106,841 | - |

*Abbreviations: SD=Standard Deviation.*

## Table S2.3: Covariate availability across the total sample (n=4,317,436).

|  | **Missing, n (%)** | **Available, n (%)** |
| --- | --- | --- |
| Gender | 95,788 (2.2%) | 4,221,648 (97.8%) |
| Ethnicity | 98,820 (2.3%) | 4,218,616 (97.7%) |
| FSM eligibility | 95,793 (2.2%) | 4,221,643 (97.8%) |
| SEN provision | 95,788 (2.2%) | 4,221,648 (97.8%) |
| Local authority | 95,788 (2.2%) | 4,221,648 (97.8%) |
| School | 95,788 (2.2%) | 4,221,648 (97.8%) |

## Table S2.4: Characteristics of complete versus incomplete cases on fully available variables

|  | **Incomplete cases,**  **n (%)**  **(n=98,825)** | **Complete cases,**  **n (%)**  **(n=4,218,611)** | **Full sample,**  **n (%)**  **(n=4,317,436)** |
| --- | --- | --- | --- |
| Trajectory membership |  |  |  |
| *Average/High Consistent* | 94,657 (95.8%) | 3,402,510 (80.7%) | 3,497,167 (81.0%) |
| *Average/High Increasing* | 338 (0.3%) | 66,045 (1.6%) | 66,383 (1.5%) |
| *Average Declining* | 2,798 (2.8%) | 370,319 (8.8%) | 373,117 (8.6%) |
| *Low Increasing* | 237 (0.2%) | 98,568 (2.3%) | 98,805 (2.3%) |
| *Low Consistent* | 795 (0.8%) | 281,169 (6.7%) | 281,964 (6.5%) |
| Any first offence conviction or caution during young adulthood |  |  |  |
| *No* | 96,546 (97.7%) | 4,007,675 (95.0%) | 4,104,221 (95.1%) |
| *Yes* | 2,279 (2.3%) | 210,936 (5.0%) | 213,215 (4.9%) |
| First offence conviction or caution during young adulthood for serious violence |  |  |  |
| *No* | 98,715 (99.9%) | 4,199,594 (99.6%) | 4,298,309 (99.6%) |
| *Yes* | 110 (0.1%) | 19,017 (0.5%) | 19,127 (0.4%) |
| LAC status in Year 11 |  |  |  |
| *Not LAC* | 98,740 (k) | 4,168,510 (99.0%) | 4,267,250 (99.0%) |
| *LAC* | 80 (k) | 50,100 (1.0%) | 50,180 (1.0%) |
| Year 11 assessment year (median, IQR) | 2008 (2007 to 2010) | 2009 (2007 to 2011) | 2009 (2007 to 2011) |

*Note: In accordance with Children Looked After statistical disclosure control guidelines, LAC status frequencies have been rounded to the nearest 10, and percentages to the nearest integer. ‘k’ is used when a result that is not 100%/0% would appear as such due to rounding.*

*Abbreviations: LAC=Looked After Child; IQR=Interquartile Range.*

# Supplement 3 – Supporting information for trajectory modelling findings

## Figure S3.1: Trajectory shapes for LGMM solutions with one to five latent trajectories.

## Table S3.2: Unstandardised parameter estimates for the five-trajectory solution.

|  | **Intercept** | | **Slope** | |
| --- | --- | --- | --- | --- |
|  | **Mean** | **95% CI** | **Mean** | **95% CI** |
| **Average/High Consistent (n=3,497,167)** | 0.27 | 0.27 to 0.27 | -0.00 | -0.00 to -0.00 |
| **Average/High Increasing (n=66,383)** | 0.12 | 0.12 to 0.13 | 0.16 | 0.16 to 0.16 |
| **Average Declining (n=373,117)** | -0.23 | -0.23 to -0.22 | -0.14 | -0.14 to -0.14 |
| **Low Increasing (n=98,805)** | -2.08 | -2.09 to -2.08 | 0.23 | 0.23 to 0.24 |
| **Low Consistent (n=281,964)** | -2.19 | -2.19 to -2.18 | 0.08 | 0.08 to 0.08 |

*Note: Symmetric 95% confidence intervals are reported. The intercept estimates correspond to Year 2. Residual variances were held equal across trajectories. In each trajectory, residual variance was 0.05 at Year 2, 0.31 at Year 6, and 0.29 at Year 11.*

*Abbreviations: CI=Confidence Interval*.

## Table S3.3: Proportion of pupils meeting expected attainment thresholds for each school year in each trajectory, n (%).

|  | **Year 2** | **Year 6** | **Year 11** |
| --- | --- | --- | --- |
| **Average/High Consistent (n=3,497,167)** | 86.9% | 79.9% | 63.9% |
| **Average/High Increasing (n=66,383)** | 86.4% | 95.1% | 93.5% |
| **Average Declining (n=373,117)** | 63.0% | 29.7% | 0.7% |
| **Low Increasing (n=98,805)** | <0.1% | 20.0% | 21.1% |
| **Low Consistent (n=281,964)** | <0.1% | 1.5% | 0.4% |

*Note: In Year 2, this is the proportion achieving a Level 2 or above in reading, writing, maths and science assessments. In Year 6, this is the proportion achieving a Level 4 or above in English and maths assessments. In Year 11, this is the proportion achieving 5 A* to C GCSE or equivalent grades including English and maths.*

# Supplement 4 – Supporting information for results

## Table S4.1: Sample characteristics stratified by attainment trajectory membership.

|  | **Attainment trajectory membership** | | | | |
| --- | --- | --- | --- | --- | --- |
|  | **Average/High Consistent**  **(n=3,402,510)** | **Average/High Increasing**  **(n=66,045)** | **Average Declining**  **(n=370,319)** | **Low Increasing (n=98,568)** | **Low Consistent**  **(n=281,169)** |
| Gender |  |  |  |  |  |
| *Female* | 1,723,075 | 38,373 | 150,221 | 43,377 | 105,189 |
|  | (50.6%) | (58.1%) | (40.6%) | (44.0%) | (37.4%) |
| *Male* | 1,679,435 | 27,672 | 220,098 | 55,191 | 175,980 |
|  | (49.4%) | (41.9%) | (59.4%) | (56.0%) | (62.6%) |
| Ethnic group |  |  |  |  |  |
| *White* | 2,901,884 | 51,496 | 326,500 | 64,940 | 229,666 |
|  | (85.3%) | (78.0%) | (88.2%) | (65.9%) | (81.7%) |
| *Black* | 122,100 | 2,203 | 13,715 | 8,834 | 15,034 |
|  | (3.6%) | (3.3%) | (3.7%) | (9.0%) | (5.4%) |
| *Asian* | 242,548 | 9,072 | 13,731 | 18,241 | 24,371 |
|  | (7.1%) | (13.7%) | (3.7%) | (18.5%) | (8.7%) |
| *Mixed* | 107,346 | 2,164 | 14,200 | 3,409 | 8,692 |
|  | (3.2%) | (3.3%) | (3.8%) | (3.5%) | (3.1%) |
| *Other* | 28,632 | 1,110 | 2,173 | 3,144 | 3,406 |
|  | (0.8%) | (1.7%) | (0.6%) | (3.2%) | (1.2%) |
| FSM eligibility |  |  |  |  |  |
| *Ineligible* | 3,059,375 | 59,646 | 273,101 | 71,058 | 190,947 |
|  | (89.9%) | (90.3%) | (73.8%) | (72.1%) | (67.9%) |
| *Eligible* | 343,135 | 6,399 | 97,218 | 27,510 | 90,222 |
|  | (10.1%) | (9.7%) | (26.3%) | (27.9%) | (32.1%) |
| LAC status in Year 11 |  |  |  |  |  |
| *Not LAC* | 3,386,710 | 65,920 | 354,430 | 96,690 | 264,760 |
|  | (k) | (k) | (96.0%) | (98.0%) | (94.0%) |
| *LAC* | 15,800 | 130 | 15,890 | 1,880 | 16,410 |
|  | (k) | (k) | (4.0%) | (2.0%) | (6.0%) |
| SEN provision |  |  |  |  |  |
| *None* | 2,919,060 | 61,350 | 159,149 | 48,456 | 52,254 |
|  | (85.8%) | (92.9%) | (43.0%) | (49.2%) | (18.6%) |
| *AAP/S* | 453,298 | 4,443 | 181,293 | 40,506 | 131,140 |
|  | (13.3%) | (6.7%) | (49.0%) | (41.1%) | (46.6%) |
| *S/EHCPS* | 30,152 | 252 | 29,877 | 9,606 | 97,775 |
|  | (0.9%) | (0.4%) | (8.1%) | (9.8%) | (34.8%) |
| Year 11 assessment year (median, IQR) | 2009 (2007 to 2011) | 2009 (2007 to 2011) | 2009 (2007 to 2011) | 2009 (2007 to 2011) | 2009 (2007 to 2011) |

*Note: In accordance with Children Looked After statistical disclosure control guidelines, LAC status frequencies have been rounded to the nearest 10, and percentages to the nearest integer.*

*Abbreviations: AAP/S=action, action plus or support; FSM=Free School Meal eligibility; LAC=Looked After Child; IQR=Interquartile Range; S/EHCPS=statement of SEN or an education, health and care plan; SEN=Special Educational Needs.*

## Table S4.2: Single-level, variance-components, and multilevel models, with the outcome as any first offence conviction or caution during young adulthood (primary outcome).

|  | **Model 1**  **Odds (95% CI)** | **Model 2**  **Odds (95% CI)** | **Model 3**  **Odds (95% CI)** | **Model 4**  **OR (95% CI)** | **Model 5**  **OR (95% CI)** | **Model 6**  **OR (95% CI)** |
| --- | --- | --- | --- | --- | --- | --- |
| **Fixed effects parameter estimates** |  |  |  |  |  |  |
| Intercept | 0.05 (0.05 to 0.05) | 0.05 (0.04 to 0.05) | 0.05 (0.05 to 0.05) | 0.04 (0.04 to 0.04) | 0.04 (0.04 to 0.04) | 0.04 (0.04 to 0.04) |
| Attainment trajectory membership |  |  |  |  |  |  |
| *Average/High Consistent* | - | - | - | Reference | Reference | Reference |
| *Average/High Increasing* | - | - | - | 0.33 (0.31 to 0.35) | 0.33 (0.31 to 0.35) | 0.35 (0.33 to 0.38) |
| *Average Declining* | - | - | - | 2.30 (2.27 to 2.33) | 2.30 (2.27 to 2.33) | 2.46 (2.43 to 2.49) |
| *Low Increasing* | - | - | - | 1.18 (1.15 to 1.22) | 1.18 (1.15 to 1.22) | 1.34 (1.30 to 1.38) |
| *Low Consistent* | - | - | - | 2.04 (2.01 to 2.07) | 2.04 (2.01 to 2.07) | 2.11 (2.08 to 2.14) |
| **Random effects parameter estimates** |  |  |  |  |  |  |
| *School variance* | - | 0.21 (0.20 to 0.22) | 0.19 (0.18 to 0.20) | 0.15 (0.14 to 0.16) | 0.17 (0.16 to 0.18) | - |
| *LA variance* | - | - | 0.02 (0.01 to 0.03) | 0.02 (0.01 to 0.02) | - | - |
| **ICC** |  |  |  |  |  |  |
| *School ICC* | - | 5.9% | 5.9% | 4.7% | 4.8% | - |
| *LA ICC* | - | - | 0.6% | 0.5% | - | - |
| **Deviance** | 1,674,949 | 1,656,360 | 1,656,547 | 1,634,112 | 1,634,204 | 1,647,736 |

*Notes: Pupil n=4,218,611, school n=19,554, LA n=154. Model 1: Single-level logistic regression model containing outcome variable only. Model 2: Two-level variance components model including school as a level 2 clustering variable. Model 3: Three-level variance components model including school as a level 2 clustering variable, and LA as a level 3 clustering variable. Model 4: Three-level multilevel model including attainment trajectory as a level 1 covariate (random intercept), school as a level 2 clustering variable, and LA as a level 3 clustering variable. Model 5: Two-level multilevel model including attainment trajectory as a level 1 covariate (random intercept) and school as a level 2 clustering variable. Model 6: Single-level logistic regression model including attainment trajectory as a level 1 covariate (random intercept).*

*Abbreviations: CI=confidence interval; ICC=intra class correlation; LA=local authority; OR=odds ratio.*

## Table S4.3: Single-level, variance-components, and multilevel models, with the outcome as first offence conviction or caution during young adulthood for serious violence (secondary outcome).

|  | **Model 1**  **Odds (95% CI)** | **Model 2**  **Odds (95% CI)** | **Model 3**  **Odds (95% CI)** | **Model 4**  **OR (95% CI)** | **Model 5**  **OR (95% CI)** | **Model 6**  **OR (95% CI)** |
| --- | --- | --- | --- | --- | --- | --- |
| **Fixed effects parameter estimates** |  |  |  |  |  |  |
| Intercept | 0.01 (0.00 to 0.01) | 0.00 (0.00 to 0.00) | 0.00 (0.00 to 0.00) | 0.00 (0.00 to 0.00) | 0.00 (0.00 to 0.00) | 0.00 (0.00 to 0.00) |
| Attainment trajectory membership |  |  |  |  |  |  |
| *Average/High Consistent* | - | - | - | Reference | Reference | Reference |
| *Average/High Increasing* | - | - | - | 0.26 (0.20 to 0.33) | 0.26 (0.21 to 0.33) | 0.29 (0.23 to 0.37) |
| *Average Declining* | - | - | - | 2.53 (2.43 to 2.63) | 2.50 (2.41 to 2.60) | 2.70 (2.60 to 2.80) |
| *Low Increasing* | - | - | - | 1.33 (1.22 to 1.45) | 1.36 (1.25 to 1.48) | 1.71 (1.57 to 1.85) |
| *Low Consistent* | - | - | - | 2.27 (2.17 to 2.37) | 2.29 (2.19 to 2.39) | 2.52 (2.42 to 2.64) |
| **Random effects parameter estimates** |  |  |  |  |  |  |
| *School variance* | - | 0.38 (0.35 to 0.41) | 0.22 (0.20 to 0.25) | 0.17 (0.15 to 0.19) | 0.32 (0.29 to 0.35) | - |
| *LA variance* | - | - | 0.12 (0.09 to 0.16) | 0.12 (0.09 to 0.15) | - | - |
| **ICC** |  |  |  |  |  |  |
| *School ICC* | - | 10.4% | 9.5% | 8.1% | 8.8% | - |
| *LA ICC* | - | - | 3.4% | 3.2% | - | - |
| **Deviance** | 243,405 | 240,146 | 239,619 | 236,663 | 237,228 | 239,792 |

*Notes: Pupil n=4,218,611, school n=19,554, LA n=154. Model 1: Single-level logistic regression model containing outcome variable only. Model 2: Two-level variance components model including school as a level 2 clustering variable. Model 3: Three-level variance components model including school as a level 2 clustering variable, and LA as a level 3 clustering variable. Model 4: Three-level multilevel model including attainment trajectory as a level 1 covariate (random intercept), school as a level 2 clustering variable, and LA as a level 3 clustering variable. Model 5: Two-level multilevel model including attainment trajectory as a level 1 covariate (random intercept) and school as a level 2 clustering variable. Model 6: Single-level logistic regression model including attainment trajectory as a level 1 covariate (random intercept).*

*Abbreviations: CI=confidence interval; ICC=intra class correlation; LA=local authority; OR=odds ratio.*

## Table S4.4: Interaction terms between attainment trajectory membership and each sociodemographic covariate, with the outcome variable as any first offence conviction or caution during young adulthood.

| **Interaction term** | **Fully adjusted**  **OR (95% CI)** |
| --- | --- |
| Gender x Attainment trajectory membership |  |
| *Male x Average/High Increasing* | 1.16 (1.00 to 1.34) |
| *Male x Average Declining* | 0.55 (0.53 to 0.56) |
| *Male x Low Increasing* | 0.98 (0.91 to 1.05) |
| *Male x Low Consistent* | 0.65 (0.63 to 0.67) |
| Ethnicity x Attainment trajectory membership |  |
| *Black x Average/High Increasing* | 1.04 (0.78 to 1.37) |
| *Black x Average Declining* | 0.71 (0.67 to 0.75) |
| *Black x Low Increasing* | 0.85 (0.78 to 0.94) |
| *Black x Low Consistent* | 0.78 (0.74 to 0.83) |
| *Asian x Average/High Increasing* | 0.96 (0.78 to 1.16) |
| *Asian x Average Declining* | 1.13 (1.06 to 1.20) |
| *Asian x Low Increasing* | 0.91 (0.84 to 0.99) |
| *Asian x Low Consistent* | 0.93 (0.88 to 0.99) |
| *Mixed x Average/High Increasing* | 0.95 (0.69 to 1.31) |
| *Mixed x Average Declining* | 0.75 (0.70 to 0.79) |
| *Mixed x Low Increasing* | 1.00 (0.87 to 1.14) |
| *Mixed x Low Consistent* | 0.83 (0.77 to 0.90) |
| *Other x Average/High Increasing* | 1.30 (0.85 to 1.98) |
| *Other x Average Declining* | 0.91 (0.78 to 1.06) |
| *Other x Low Increasing* | 1.06 (0.90 to 1.25) |
| *Other x Low Consistent* | 0.95 (0.83 to 1.09) |
| FSM eligibility x Attainment trajectory membership |  |
| *Eligible x Average/High Increasing* | 1.11 (0.93 to 1.32) |
| *Eligible x Average Declining* | 0.66 (0.64 to 0.68) |
| *Eligible x Low Increasing* | 0.83 (0.78 to 0.88) |
| *Eligible x Low Consistent* | 0.77 (0.75 to 0.80) |
| LAC status in Year 11 x Attainment trajectory membership |  |
| *LAC x Average/High Increasing* | * |
| *LAC x Average Declining* | 0.36 (0.33 to 0.39) |
| *LAC x Low Increasing* | 1.18 (1.01 to 1.39) |
| *LAC x Low Consistent* | 0.52 (0.48 to 0.57) |
| SEN provision x Attainment trajectory membership |  |
| *AAP/S x Average/High Increasing* | 1.02 (0.84 to 1.24) |
| *AAP/S x Average Declining* | 0.58 (0.56 to 0.59) |
| *AAP/S x Low Increasing* | 0.74 (0.70 to 0.78) |
| *AAP/S x Low Consistent* | 0.61 (0.59 to 0.63) |
| *S/EHCPS x Average/High Increasing* | * |
| *S/EHCPS x Average Declining* | 0.62 (0.58 to 0.66) |
| *S/EHCPS x Low Increasing* | 0.83 (0.75 to 0.93) |
| *S/EHCPS x Low Consistent* | 0.61 (0.57 to 0.65) |

*Notes: Interactions between attainment trajectory membership and each sociodemographic covariate were added one at a time to the fully adjusted multilevel logistic regression model in Table 4 of the main manuscript. Pupil n=4,218,611, school n=19,554.*

** Suppressed due to small underlying cell counts.*

*Abbreviations: AAP/S=action, action plus or support; CI=confidence interval; FSM=Free School Meal eligibility; LAC=Looked After Child; OR=odds ratio; S/EHCPS=statement of SEN or an education, health and care plan; SEN=Special Educational Needs.*

## Table S4.5: Multilevel logistic regression models stratified by attainment trajectory membership, fully adjusted for all remaining covariates, and with the outcome as any first offence conviction or caution during young adulthood (primary outcome).

| **Fixed effects parameter estimates** | **OR (95% CI) in Average/High Consistent group^a^** | **OR (95% CI) in Average/High Increasing group^b^** | **OR (95% CI) in Average Declining group^c^** | **OR (95% CI) in Low Increasing group^d^** | **OR (95% CI) in Low Consistent group^e^** |
| --- | --- | --- | --- | --- | --- |
| Intercept | 0.03 (0.03 to 0.03) | 0.01 (0.01 to 0.01) | 0.08 (0.08 to 0.08) | 0.03 (0.03 to 0.03) | 0.07 (0.07 to 0.08) |
| Gender |  |  |  |  |  |
| *Female* | Ref | Ref | Ref | Ref | Ref |
| *Male* | 3.75 (3.70 to 3.80) | 4.39 (3.79 to 5.09) | 2.09 (2.04 to 2.14) | 3.78 (3.52 to 4.05) | 2.49 (2.41 to 2.57) |
| Ethnic group |  |  |  |  |  |
| *White* | Ref | Ref | Ref | Ref | Ref |
| *Black* | 1.58 (1.54 to 1.62) | 1.64 (1.22 to 2.20) | 1.23 (1.16 to 1.30) | 1.43 (1.31 to 1.57) | 1.26 (1.19 to 1.33) |
| *Asian* | 0.86 (0.84 to 0.88) | 0.80 (0.65 to 0.98) | 0.99 (0.94 to 1.05) | 0.79 (0.72 to 0.85) | 0.79 (0.74 to 0.83) |
| *Mixed* | 1.41 (1.38 to 1.45) | 1.32 (0.96 to 1.82) | 1.09 (1.03 to 1.15) | 1.41 (1.23 to 1.61) | 1.18 (1.10 to 1.28) |
| *Other* | 0.98 (0.92 to 1.03) | 1.23 (0.79 to 1.89) | 0.93 (0.80 to 1.08) | 1.09 (0.93 to 1.27) | 0.92 (0.81 to 1.04) |
| FSM eligibility |  |  |  |  |  |
| *Ineligible* | Ref | Ref | Ref | Ref | Ref |
| *Eligible* | 1.54 (1.52 to 1.57) | 1.77 (1.48 to 2.13) | 1.05 (1.02 to 1.07) | 1.37 (1.29 to 1.46) | 1.23 (1.20 to 1.27) |
| LAC status in Year 11 |  |  |  |  |  |
| *Not LAC* | Ref | Ref | Ref | Ref | Ref |
| *LAC* | 1.93 (1.82 to 2.04) | * | 0.72 (0.68 to 0.77) | 2.44 (2.09 to 2.84) | 1.05 (0.99 to 1.12) |
| SEN provision |  |  |  |  |  |
| *None* | Ref | Ref | Ref | Ref | Ref |
| *AAP/S* | 1.62 (1.60 to 1.64) | 1.57 (1.28 to 1.92) | 1.01 (0.99 to 1.04) | 1.20 (1.13 to 1.27) | 1.01 (0.98 to 1.05) |
| *S/EHCPS* | 1.01 (0.96 to 1.06) | * | 0.73 (0.70 to 0.76) | 0.83 (0.75 to 0.91) | 0.65 (0.62 to 0.67) |
| Year 11 assessment year | 0.86 (0.86 to 0.86) | 0.87 (0.85 to 0.90) | 0.95 (0.94 to 0.95) | 0.88 (0.87 to 0.90) | 0.91 (0.90 to 0.91) |
| **Random effect: school variance** | 0.06 (0.05 to 0.06) | 0.22 (0.13 to 0.36) | 0.03 (0.02 to 0.03) | 0.04 (0.02 to 0.08) | 0.06 (0.05 to 0.08) |
| **School ICC** | 1.7% | 6.2% | 0.8% | 1.1% | 1.8% |
| **Deviance** | 1,113,859 | 9797 | 233,048 | 40,164 | 158,603 |

*Notes: ^a^ Pupil n=3,402,510, school n=18,222. ^b^ Pupil n=66,045, school n=3,416. ^c^ Pupil n=370,319, school n=8,436. ^d^ Pupil n=98,568, school n=4,506. ^e^ Pupil n=281,169, school n=6,898. It should be noted that cell counts underlying stratified analyses are small, and should be interpreted with caution.*

** Suppressed due to small underlying cell counts.*

*Abbreviations: AAP/S=action, action plus or support; CI=confidence interval; FSM=Free School Meal eligibility; ICC=intra class correlation; LAC=Looked After Child; OR=odds ratio; S/EHCPS=statement of SEN or an education, health and care plan; SEN=Special Educational Needs.*

## Table S4.6: Two-level multilevel logistic regression models with random slopes for attainment trajectory membership at the level of local authorities, with the outcome as any first offence conviction or caution during young adulthood (primary outcome).

|  | **Random intercept model**  **OR (95% CI)** | **Random slope on Average/High Consistent trajectory**  **OR (95% CI)** | **Random slope on Average/High Increasing trajectory**  **OR (95% CI)** | **Random slope on Average Declining trajectory**  **OR (95% CI)** | **Random slope on Low Increasing trajectory**  **OR (95% CI)** | **Random slope on Low Consistent trajectory**  **OR (95% CI)** |
| --- | --- | --- | --- | --- | --- | --- |
| **Fixed effects parameter estimates** |  |  |  |  |  |  |
| Intercept | 0.05 (0.04 to 0.05) | 0.05 (0.04 to 0.05) | 0.05 (0.04 to 0.05) | 0.05 (0.04 to 0.05) | 0.05 (0.04 to 0.05) | 0.05 (0.04 to 0.05) |
| Attainment trajectory membership |  |  |  |  |  |  |
| *Average/High Consistent* | Reference | Reference | Reference | Reference | Reference | Reference |
| *Average/High Increasing* | 0.28 (0.23 to 0.34) | 0.28 (0.23 to 0.34) | 0.28 (0.23 to 0.35) | 0.28 (0.23 to 0.34) | 0.28 (0.23 to 0.34) | 0.28 (0.23 to 0.34) |
| *Average Declining* | 2.46 (2.38 to 2.54) | 2.45 (2.30 to 2.61) | 2.46 (2.38 to 2.54) | 2.42 (2.25 to 2.59) | 2.46 (2.38 to 2.54) | 2.46 (2.38 to 2.54) |
| *Low Increasing* | 1.37 (1.28 to 1.47) | 1.37 (1.26 to 1.50) | 1.37 (1.28 to 1.47) | 1.36 (1.27 to 1.46) | 1.37 (1.27 to 1.49) | 1.37 (1.27 to 1.47) |
| *Low Consistent* | 2.09 (2.01 to 2.17) | 2.08 (1.95 to 2.23) | 2.09 (2.01 to 2.17) | 2.09 (2.01 to 2.17) | 2.09 (2.01 to 2.17) | 2.09 (1.99 to 2.20) |
| **Random effects parameter estimates** |  |  |  |  |  |  |
| *LA variance* | 0.04 (0.02 to 0.07) | 0.02 (0.01 to 0.05) | 0.04 (0.02 to 0.07) | 0.04 (0.02 to 0.08) | 0.04 (0.02 to 0.07) | 0.04 (0.02 to 0.08) |
| *Trajectory variance* | - | 0.01 (0.01 to 0.03) | 0.03 (0.00 to 1.18) | 0.02 (0.01 to 0.04) | 0.00 (0.00 to 0.18) | 0.00 (0.00 to 0.02) |
| *Trajectory / LA covariance* | - | 0.01 (-0.00 to 0.01) | 0.02 (-0.02 to 0.07) | -0.02 (-0.04 to -0.00) | -0.00 (-0.02 to 0.01) | -0.01 (-0.02 to 0.00) |
| **LA ICC** | 1.1% | 0.7% | 1.1% | 1.3% | 1.1% | 1.2% |
| **Deviance** | 240,326 | 240,278 | 240,332 | 240,289 | 240,333 | 240,328 |

*Notes: Analysis restricted to a random n=20 local authorities. Pupil n=620,626, LA n=20.*

*Abbreviations: CI=confidence interval; ICC=intra class correlation; LA=local authority; OR=odds ratio.*

## Table S4.7: Two-level multilevel logistic regression models with random slopes for attainment trajectory membership at the level of schools, with the outcome as any first offence conviction or caution during young adulthood (primary outcome).

|  | **Random intercept model**  **OR (95% CI)** | **Random slope on Average/High Consistent trajectory**  **OR (95% CI)** | **Random slope on Average/High Increasing trajectory**  **OR (95% CI)** | **Random slope on Average Declining trajectory**  **OR (95% CI)** | **Random slope on Low Increasing trajectory**  **OR (95% CI)** | **Random slope on Low Consistent trajectory**  **OR (95% CI)** |
| --- | --- | --- | --- | --- | --- | --- |
| **Fixed effects parameter estimates** |  |  |  |  |  |  |
| Intercept | 0.04 (0.03 to 0.04) | 0.04 (0.03 to 0.04) | 0.04 (0.03 to 0.04) | 0.04 (0.03 to 0.04) | 0.04 (0.03 to 0.04) | 0.04 (0.03 to 0.04) |
| Attainment trajectory membership |  |  |  |  |  |  |
| *Average/High Consistent* | Reference | Reference | Reference | Reference | Reference | Reference |
| *Average/High Increasing* | 0.26 (0.22 to 0.32) | 0.30 (0.24 to 0.36) | 0.24 (0.18 to 0.33) | 0.26 (0.22 to 0.32) | 0.26 (0.22 to 0.32) | 0.26 (0.22 to 0.32) |
| *Average Declining* | 2.29 (2.22 to 2.37) | 2.56 (2.44 to 2.68) | 2.29 (2.22 to 2.37) | 2.69 (2.56 to 2.82) | 2.29 (2.22 to 2.37) | 2.29 (2.22 to 2.37) |
| *Low Increasing* | 1.22 (1.14 to 1.32) | 1.41 (1.30 to 1.52) | 1.22 (1.14 to 1.32) | 1.20 (1.12 to 1.29) | 1.22 (1.08 to 1.38) | 1.22 (1.14 to 1.31) |
| *Low Consistent* | 2.05 (1.97 to 2.14) | 2.27 (2.16 to 2.39) | 2.05 (1.97 to 2.14) | 2.03 (1.95 to 2.12) | 2.05 (1.97 to 2.14) | 2.09 (1.98 to 2.22) |
| **Random effects parameter estimates** |  |  |  |  |  |  |
| *School variance* | 0.21 (0.18 to 0.24) | 0.12 (0.09 to 0.15) | 0.21 (0.18 to 0.24) | 0.24 (0.21 to 0.28) | 0.21 (0.18 to 0.24) | 0.21 (0.18 to 0.25) |
| *Trajectory variance* | - | 0.05 (0.04 to 0.08) | 0.01 (0.00 to 1.45) | 0.08 (0.05 to 0.12) | 0.02 (0.00 to 0.81) | 0.04 (0.02 to 0.09) |
| *Trajectory / School covariance* | - | 0.04 (0.03 to 0.06) | 0.04 (-0.07 to 0.15) | -0.13 (-0.16 to -0.10) | -0.00 (-0.05 to 0.04) | -0.03 (-0.06 to -0.00) |
| **School ICC** | 5.9% | 3.5% | 5.9% | 6.8% | 5.9% | 6.0% |
| **Deviance** | 238,659 | 238,547 | 238,660 | 238,535 | 238,660 | 238,648 |

*Notes: Analysis restricted to a random n=20 local authorities. Pupil n=620,626, School n=2,838.*

*Abbreviations: CI=confidence interval; ICC=intra class correlation; OR=odds ratio.*

## Table S4.8: Sensitivity multilevel logistic regression analysis showing adjusted associations between attainment trajectory membership and any first offence conviction or caution during young adulthood (primary outcome), excluding pupils who were convicted or cautioned for an earlier offence before the end of secondary school from the reference group.

|  | **Fully adjusted**  **OR (95% CI)** |
| --- | --- |
| **Fixed effects parameter estimates** |  |
| Intercept | 0.03 (0.03 to 0.03) |
| Attainment trajectory membership |  |
| *Average/High Consistent* | Reference |
| *Average/High Increasing* | 0.36 (0.34 to 0.39) |
| *Average Declining* | 2.78 (2.74 to 2.82) |
| *Low Increasing* | 0.97 (0.94 to 0.99) |
| *Low Consistent* | 1.71 (1.68 to 1.74) |
| **Random effect: school variance** | 0.10 (0.09 to 0.10) |
| **School ICC** | 2.8% |
| **Deviance** | 1,477,533 |

*Notes: Pupil n=3,849,054, school n=19,315.*

*Abbreviations: CI=confidence interval; ICC=intra class correlation; OR=odds ratio. Adjusted for gender, ethnicity, FSM eligibility, LAC status in Year 11, SEN provision, and Year 11 assessment year.*

## Table S4.9: Sensitivity multilevel logistic regression models stratified by attainment trajectory membership, fully adjusted for all remaining covariates, and with the outcome as any first offence conviction or caution during young adulthood (primary outcome), excluding pupils who were convicted or cautioned for an earlier offence before the end of secondary school from the reference group.

| **Fixed effects parameter estimates** | **OR (95% CI) in Average/High Consistent group^a^** | **OR (95% CI) in Average/High Increasing group^b^** | **OR (95% CI) in Average Declining group^c^** | **OR (95% CI) in Low Increasing group^d^** | **OR (95% CI) in Low Consistent group^e^** |
| --- | --- | --- | --- | --- | --- |
| Intercept | 0.03 (0.03 to 0.03) | 0.01 (0.01 to 0.01) | 0.12 (0.11 to 0.12) | 0.03 (0.03 to 0.04) | 0.09 (0.08 to 0.09) |
| Gender |  |  |  |  |  |
| *Female* | Ref | Ref | Ref | Ref | Ref |
| *Male* | 3.94 (3.89 to 3.99) | 4.45 (3.84 to 5.15) | 2.89 (2.81 to 2.96) | 4.04 (3.77 to 4.34) | 3.04 (2.94 to 3.14) |
| Ethnic group |  |  |  |  |  |
| *White* | Ref | Ref | Ref | Ref | Ref |
| *Black* | 1.62 (1.58 to 1.67) | 1.68 (1.25 to 2.25) | 1.33 (1.25 to 1.41) | 1.45 (1.32 to 1.59) | 1.24 (1.17 to 1.32) |
| *Asian* | 0.83 (0.81 to 0.85) | 0.79 (0.64 to 0.97) | 0.82 (0.77 to 0.87) | 0.75 (0.69 to 0.81) | 0.67 (0.63 to 0.71) |
| *Mixed* | 1.48 (1.44 to 1.53) | 1.34 (0.97 to 1.84) | 1.31 (1.23 to 1.39) | 1.44 (1.25 to 1.65) | 1.32 (1.22 to 1.42) |
| *Other* | 0.95 (0.89 to 1.01) | 1.21 (0.78 to 1.86) | 0.82 (0.71 to 0.96) | 1.05 (0.90 to 1.23) | 0.80 (0.70 to 0.91) |
| FSM eligibility |  |  |  |  |  |
| *Ineligible* | Ref | Ref | Ref | Ref | Ref |
| *Eligible* | 1.70 (1.67 to 1.73) | 1.82 (1.52 to 2.19) | 1.28 (1.25 to 1.31) | 1.47 (1.38 to 1.56) | 1.41 (1.37 to 1.46) |
| LAC status in Year 11 |  |  |  |  |  |
| *Not LAC* | Ref | Ref | Ref | Ref | Ref |
| *LAC* | 3.13 (2.95 to 3.32) | * | 1.96 (1.82 to 2.10) | 3.15 (2.69 to 3.68) | 1.51 (1.41 to 1.61) |
| SEN provision |  |  |  |  |  |
| *None* | Ref | Ref | Ref | Ref | Ref |
| *AAP/S* | 1.82 (1.80 to 1.85) | 1.62 (1.32 to 1.98) | 1.21 (1.18 to 1.24) | 1.24 (1.17 to 1.31) | 1.09 (1.05 to 1.13) |
| *S/EHCPS* | 1.10 (1.04 to 1.16) | * | 0.80 (0.76 to 0.84) | 0.84 (0.76 to 0.93) | 0.63 (0.60 to 0.66) |
| Year 11 assessment year | 0.84 (0.84 to 0.85) | 0.87 (0.84 to 0.90) | 0.90 (0.89 to 0.90) | 0.87 (0.86 to 0.88) | 0.87 (0.87 to 0.88) |
| **Random effect: school variance** | 0.07 (0.07 to 0.08) | 0.23 (0.14 to 0.37) | 0.03 (0.02 to 0.04) | 0.04 (0.02 to 0.08) | 0.13 (0.11 to 0.15) |
| **School ICC** | 2.1% | 6.5% | 0.9% | 1.2% | 3.8% |
| **Deviance** | 1,086,245 | 9,744 | 195,081 | 39,041 | 144,922 |

*Notes: ^a^ Pupil n=3,221,050, school n=18,097. ^b^ Pupil n=64,923, school n=3,411. ^c^ Pupil n=242,715, school n=7,942. ^d^ Pupil n=92,039, school n=4,409. ^e^ Pupil n=228,327, school n=6,525. It should be noted that cell counts underlying stratified analyses are small, and should be interpreted with caution.*

** Odds ratio suppressed due to underlying cell counts n<10.*

*Abbreviations: AAP/S=action, action plus or support; CI=confidence interval; FSM=Free School Meal eligibility; ICC=intra class correlation; LAC=Looked After Child; OR=odds ratio; S/EHCPS=statement of SEN or an education, health and care plan; SEN=Special Educational Needs.*

## Table S4.10: Proportion of pupils in each attainment trajectory who were convicted or cautioned for different types of first offence during young adulthood.

|  | **Attainment trajectory membership** | | | | |
| --- | --- | --- | --- | --- | --- |
| **Offence type** | **Average/High Consistent**  **(n=3,402,510)** | **Average/High Increasing**  **(n=66,045)** | **Average Declining**  **(n=370,319)** | **Low Increasing (n=98,568)** | **Low Consistent**  **(n=281,169)** |
| Violence against the person | 0.3% | 0.1% | 0.6% | 0.4% | 0.5% |
| Sexual offences | 0.1% | <0.1% | 0.1% | 0.1% | 0.2% |
| Robbery | 0.1% | <0.1% | 0.2% | 0.1% | 0.2% |
| Theft offences | 0.7% | 0.2% | 1.8% | 1.1% | 1.8% |
| Criminal damage and arson | 0.1% | <0.1% | 0.2% | 0.1% | 0.2% |
| Drug offences | 0.7% | 0.3% | 1.4% | 0.8% | 0.9% |
| Possession of weapons | 0.1% | 0.1% | 0.4% | 0.2% | 0.4% |
| Public order offences | 0.1% | <0.1% | 0.3% | 0.2% | 0.2% |
| Miscellaneous crimes against society | 0.1% | 0.1% | 0.2% | 0.2% | 0.3% |
| Fraud offences | 0.1% | 0.1% | 0.3% | 0.2% | 0.3% |
| Summary non-motoring | 1.7% | 0.6% | 4.6% | 2.3% | 3.8% |
| Summary motoring | 0.6% | 0.3% | 0.7% | 0.6% | 0.6% |

## Table S4.11: Number of offending days in the period among those convicted or cautioned for different types of first offence during young adulthood, stratified by attainment trajectory.

| **Number of offending days in period** | **Average/High Consistent**  **(n=144,059)** | **Average/High Improving**  **(n=1,012)** | **Average Declining**  **(n=36,332)** | **Low Improving**  **(n=5,517)** | **Low Consistent**  **(n=24,016)** |
| --- | --- | --- | --- | --- | --- |
| 1 | 97,181 | 753 | 19,030 | 3,426 | 12,906 |
|  | (67.5%) | (74.4%) | (52.4%) | (62.1%) | (53.7%) |
| 2 | 25,677 | 165 | 7,707 | 1,033 | 4,871 |
|  | (17.8%) | (16.3%) | (21.2%) | (18.7%) | (20.3%) |
| 3 | 9,702 | 54 | 3,607 | 433 | 2,308 |
|  | (6.7%) | (5.3%) | (9.9%) | (7.9%) | (9.6%) |
| 4 | 4,522 | 16 | 2,002 | 226 | 1,281 |
|  | (3.1%) | (1.6%) | (5.5%) | (4.1%) | (5.3%) |
| 5 | 2,478 | 10 | 1,206 | 125 | 737 |
|  | (1.7%) | (1.0%) | (3.3%) | (2.3%) | (3.1%) |
| >5 | 4,499 | 14 | 2,780 | 274 | 1,913 |
|  | (3.1%) | (1.4%) | (7.7%) | (5.0%) | (8.0%) |

1. Mplus User’s Guide, Chapter 8: <https://www.statmodel.com/download/usersguide/Chapter8.pdf> [↑](#footnote-ref-1)
2. Clark, S. L., & Muthén, B. (2009). Relating latent class analysis results to variables not included in the analysis. Available from: <https://www.statmodel.com/download/relatinglca.pdf> [Access date: 18 March 2026]. [↑](#footnote-ref-2)
